# Catechol-*O*-Methyltransferase Gene Val158Met Polymorphism Moderates the Effect of Social Exclusion and Inclusion on Aggression in Men: Findings From a Mixed Experimental Design

**DOI:** 10.3389/fpsyg.2020.622914

**Published:** 2021-01-26

**Authors:** Meiping Wang, Pian Chen, Hang Li, Andrew Haddon Kemp, Wenxin Zhang

**Affiliations:** ^1^School of Psychology, Shandong Normal University, Jinan, China; ^2^Department of Psychology, College of Human and Health Sciences, Swansea University, Swansea, United Kingdom

**Keywords:** COMT Val158Met polymorphism, rs4680, social exclusion and inclusion, mixed experimental design, aggression

## Abstract

Accumulating research has identified the interactive effects of catechol-*O*-methyltransferase (*COMT*) gene Val158Met polymorphism and environmental factors on aggression. However, available evidence was mainly based upon correlational design, which yields mixed findings concerning who (Val vs. Met carriers) are more affected by environmental conditions and has been challenged for the low power of analyses on gene–environment interaction. Drawing on a mixed design, we scrutinized how *COMT* Val158Met polymorphism (between-group variable) impacts on aggression, assessed by hostility, aggressive motivation, and aggressive behavior, under different social conditions (exclusion vs. inclusion, within-group variable) in a sample of 70 Chinese male undergraduate students. We found that both Val/Val homozygote and Met alleles carriers showed differences in the feelings of hostility and aggressive motivation under conditions of exclusion versus inclusion, but these differences were more pronounced for Met allele carriers. These findings implied that *COMT* Val158Met polymorphism did not respond to environmental stimuli in an all-or-none way and shed light on the importance of examining the gene–environment interaction using a mixed design.

## Introduction

Aggression is a serious and pervasive societal problem throughout the world (Anholt and Mackay, 2012). Undergraduates are going through a transition from late adolescence to early adulthood; their aggressive behavior may escalate into more serious violence (Liu et al., 2013). Given its destructive effects on an array of psychological, behavioral, and academic adjustment for both victims and aggressors (Stipek and Miles, 2008; García-Sancho et al., 2017), considerable attention has focused on the underpinnings of aggression including genetic and environmental factors (Caspi et al., 2002; Moffitt, 2005; Tuvblad and Baker, 2011; Nilsson et al., 2018). A rapidly proliferating body of research has demonstrated that the interaction of catechol-*O*-methyltransferase (*COMT*) gene and certain environments were associated with aggression (Albaugh et al., 2010; Laucht et al., 2012; Hygen et al., 2015; Zhang et al., 2016; Wang et al., 2018).

The human *COMT* gene locates on chromosome 22q11.21, which is the main metabolic enzyme of catecholamines (including epinephrine, norepinephrine, and dopamine) (Iofrida et al., 2014). The *COMT* Val158Met polymorphism (rs4680), a common biallelic single-nucleotide polymorphism (SNP) locates at codon 158, is a popular candidate polymorphism for aggression research (Anholt and Mackay, 2012). The substitution of methionine (Met) for valine (Val) results in a three-to-four-fold reduction in the activity of COMT enzyme (Albaugh et al., 2010), as a result, Met carriers have enhanced levels of dopamine in the brain, especially in the prefrontal cortex, which is more likely to trigger higher levels of aggression^1^ (Strous et al., 2003). Evidence shows that *COMT* gene Val158Met polymorphism plays a moderating role in the association between environmental factors and aggression (Bhakta et al., 2012; Qayyum et al., 2015); however, findings concerning which variants (Val vs. Met carriers) are more affected by environmental conditions remain unclear. Specifically, a broader literature has indicated that compared with Val homozygotes, Met carriers (Val/Met and Met/Met) exhibit more aggression under adverse environmental conditions but less aggression under supportive or less adverse environmental conditions (Laucht et al., 2012; Thompson et al., 2012; Zhang et al., 2016). For example, Zhang et al. (2016) found that adolescents with Met alleles showed low levels of aggression when exposed to high positive parenting but high levels of aggression when exposed to low positive parenting. However, some other work has reported that Val/Val carriers are more sensitive to the environment (Hygen et al., 2015; Tuvblad et al., 2016). Overall, there is some inconsistency regarding the interaction of *COMT* Val158Met polymorphism and aggression, with some studies showing the Met allele is the susceptibility gene and others showing the Val allele is more sensitive.

One potential explanation for these mixed results is that most of the extant gene-by-environment interaction (G × E) studies on aggression utilized a correlational design, which had major weaknesses including confounding, possible gene–environment correlation, and lower power of the analyses. Experimental design can break the potential gene–environment correlation, reduce the measurement error, and enhance the statistical power by using standard manipulation (Bakermans-Kranenburg and Van IJzendoorn, 2015). A few researchers conducted experimental designs to examine the interaction between genes and environment on human aggression (Verona et al., 2006; McDermott et al., 2009; Gallardo-Pujol et al., 2013). However, published studies are limited to between-subjects designs, which can only reveal the behavior of different genotype carriers under different environmental conditions. Such studies are deficient in accurately revealing whether the same individuals with certain genotypes behave differently under different environments, but the mixed design is sufficient in this regard. Moreover, none of these studies have focused on *COMT* gene and aggression. Therefore, the present study adopted a mixed experimental design in which individuals with different genotypes will receive repeated measurements of different experimental conditions to reveal conclusively how *COMT* gene Val158Met polymorphism interacts with environmental factors on aggression. Additionally, biological sex may underpin inconsistent findings (Verona et al., 2006; Wang and Zhang, 2010), given that boys often behave more aggressively than girls (see Frieze and Li, 2010; Björkqvist, 2018); the present study only recruited males.

Existing studies have demonstrated that social exclusion and inclusion are significantly associated with aggression (DeWall et al., 2010; Evans et al., 2015). A great deal of research supported the conclusion that social exclusion can significantly predict college students’ aggression (Twenge et al., 2001; DeBono, 2014; Riva et al., 2015). Specifically, experimental evidence showed that excluded individuals were more prone to show hostility toward others (Romero-Canyas et al., 2010) and act aggressively (Twenge and Campbell, 2003). Unlike social exclusion, social inclusion could reduce the risk of aggression; for example, DeWall et al. (2010) designed two experiments to assess the association between social inclusion and aggression, and both the results indicated that the level of aggression significantly decreased as the acceptance from others increased. Given the opposite association between social exclusion and inclusion with aggression, the present study created two environmental conditions (social exclusion as an indicator of negative environment, and social inclusion as an indicator of positive environment) by using Cyberball game, a widely used and well-validated program to induce exclusion and inclusion (Warburton et al., 2006; DeWall et al., 2010).

Briefly, in the research reported here, we sought to examine how *COMT* Val158Met polymorphism might interact with social conditions on male aggression by using a two-factor mixed experimental design (between-group variable: genotype; within-group variable: social exclusion vs. inclusion) and then answer the question, as follows: which variant (Val vs. Met carriers) is more sensitive to environmental influences? Drawing on extant research and prior findings, we hypothesized that higher levels of aggression would be displayed under the excluded condition than the included condition and that this difference would be more pronounced for carriers of Met allele of *COMT* Val158Met than for Val/Val carriers.

## Materials and Methods

### Participants and Procedure

*A priori* power analysis was conducted by using G^∗^Power (Version 3.1.9.2) (Faul et al., 2009) to determine the appropriate sample size for analysis of variance (ANOVA) with two groups, two measurement points, between- and within-factors interaction, and an expected medium effect size (Recabarren et al., 2019). A sample of 60 was obtained. Considering a possible dropout rate of 15%, 70 Asian freshmen (mean age: 18.47, *SD* = 0.90) free of neurophysiological or psychiatric illness were finally recruited from XX University in Jinan, Shandong Province of P. R. China. Self-reported data displayed that 97.14% (*N* = 68) of the sample was Chinese Han ethnicity, while 2.86% (*N* = 2) was Chinese minorities.

All participants underwent excluded and included sessions in random order separated by 1-week interval between both conditions. All sessions took place in the university laboratory and took approximately 40 min. Before each session, participants were asked to complete emotional state questionnaire, 12-item Aggression Questionnaire and a hot sauce paradigm in sequence (about 10 min).

Next, participants were told that they were going to begin the Cyberball game; they were randomly assigned to social exclusion (*N* = 35) or inclusion (*N* = 35) group to complete Cyberball game (about 15 min). After playing the game, participants were asked to complete a questionnaire about their emotional state in the game to examine whether they felt ignored or accepted (about 3 min).

Then, they completed state hostility scale, aggressive motive scale, and a hot sauce paradigm to assess aggressive behavior (about 10 min) in sequence. Upon completion, participants were asked to complete hot sauce preference to ensure the validity of assessing aggressive behavior (about 2 min).

One week later, participants were measured again, and the procedure was identical to the first session, except for condition, such that participants who were assigned to the exclusion group first session were assigned to the inclusion group at the second session, and vice versa. After the final experimental session finished, we explained to all participants that they were simply responding to the situation that we manipulate to ensure that any participant would not leave with negative feelings. Finally, participants were asked to provide their saliva sample for DNA analysis under the detailed instructions of trained investigators and received $7 for their participation. Prior to data collection, approval for questionnaire and saliva sampling was obtained from the local ethics committee, and written informed consent was obtained from participants.

### Instruments

#### Cyberball Game

The Cyberball game is a task that has been widely used to induce social exclusion and inclusion in aggression research (Williams et al., 2000; Warburton et al., 2006; DeWall et al., 2010; Hühnel et al., 2017). Participants were told that they would play a ball-throwing game with two other players who were in fact virtual computer players. During the game, they may receive the ball passed by others and could throw the ball to other players. Participants were randomly assigned to the exclusion and inclusion groups. In the exclusion condition, after receiving the ball two times at the onset of the game, the participants do not receive the balls anymore. In the inclusion condition, the participants would receive the ball 10 times of the 30 total tosses (Williams et al., 2000; Riva et al., 2015).

#### Emotional State

Participants’ emotional state was assessed by asking “how do you feel now” for one item on a 1 (very angry) to 5 (very happy) scale before the experiment in order to ensure there were no significant differences in baseline emotional state between the exclusion and inclusion groups. After the Cyberball game, participants were asked “how do you feel during the game” for one item on a 1 (very angry) to 5 (very happy) scale to assess their emotional state again to examine whether the experimental manipulation was effective.

#### Aggression

As a multifactorial construct, aggression usually refers to behaviors directed toward another individual or object carried out with the intention to harm others (Bushman and Anderson, 2001), and hostility has also been recognized as an aspect of aggression (Castillo-López et al., 2015). Therefore, besides an overall rating scale, three measurements—aggressive motivation, aggressive behavior, and hostility—were also used to better index distinct aspects of aggression in this study.

#### 12-Item Aggression Questionnaire

The baseline level of aggression prior to the experiment was measured with 12-item Aggression Questionnaire (12-AQ), adapted by Bryant and Smith (2001) and based on the Buss–Perry aggression questionnaire (BPAQ) (Buss and Perry, 1992). This questionnaire consisted of 12 items (e.g., “if someone hurt me and I’ll hit him”). Ratings for each item ranged from 1 (completely not match) to 5 (exactly match). Cronbach’s alpha values of the 12-item aggression questionnaire were 0.71 and 0.83 for two measurements (under conditions of social exclusion vs. social inclusion) in this study.

#### Hostility

Hostility was measured by state hostility scale (Anderson et al., 1995), which consisted of six items (e.g., “I want to shout to others”). Ratings for each item ranged from 1 (completely not match) to 5 (exactly match). Cronbach’s alpha values of this scale were 0.72 and 0.86 for two measurements.

#### Aggressive Motivation

Aggressive motivation was assessed by the Aggressive Motives Scale (Anderson and Murphy, 2003). The original scale consisted of six items, two of which are used to measure instrumental aggression motivation and four of which are used to measure revenge aggression motivation. Given that aggression was provoked by others in the present study, the two items used to measure instrumental aggression motivation were excluded from the scale. Sample items of revenge motivation include “I wanted to make my partner mad.” The ratings for each item ranged from 1 (not at all) to 5 (a lot). Cronbach’s alpha values for the two measurements were 0.83 and 0.89.

#### Aggressive Behavior

Aggressive behavior was assessed by a well-validated paradigm known as the hot sauce paradigm, first designed by Lieberman et al. (1999). Participants were told that their partners in Cyberball game were disgusted with hot sauce, and they had the chance to allocate the unpleasant hot sauce to punish their partners. The amount (1 to 10) of hot sauce they administrated was indicative of their aggressive behavior. Each participant responded to this paradigm before and after the Cyberball game to assess his baseline and post-experimental aggressive behavior.

#### Hot Sauce Preference

To ensure that the hot sauce allocated to their partners by participants in the hot sauce paradigm is not a function of their liking food, hot sauce preference, a well-validated measurement in the previous study (Barlett et al., 2009; Adachi and Willoughby, 2011), was assessed by asking “how much do you LIKE the hot sauce” on a 1 (not at all) to 5 (a lot) scale.

### Genotyping

DNA was isolated from saliva samples collected with Oragene collection kits (DNA Genotek, Ottawa, ON, Canada) using the Klear-gene DNA extraction method (Wang et al., 2018). SNP genotyping was performed by Shanghai Benegene Biotechnology Inc. using MassARRAY system (Agena) by means of matrix-assisted laser desorption/ionization–time of flight (MALDI-TOF) mass spectrometry method according to the manufacturer’s instructions. Briefly, the DNA sample to be queried was diluted to 5–10 ng/μl, and 1 μl of DNA was combined with 0.95 μl of water, 0.625 μl of PCR buffer containing 15 mM of MgCl_2_, 1 μl of 2.5 mM of dNTP, 0.325 μl of 25 mM MgCl_2_, 1 μl of PCR primers, and 0.1 μl of 5 units/μl HotStar Taq (Qiagen). The reaction was incubated at 94°C for 15 min followed by 45 cycles at 94°C for 20 s, 56°C for 30 s, and 72°C for 1 min, and a final incubation at 72°C for 3 min. After PCR amplification, the remaining dNTPs were dephosphorylated by adding 1.53 μl of water, 0.17 μl of SAP buffer, and 0.3 units of shrimp alkaline phosphatase (Agena). The reaction was placed at 37°C for 40 min, and the enzyme was deactivated by incubating at 85°C for 5 min. After shrimp alkaline phosphatase treatment, the single primer extension over the SNP was combined with 0.755 μl of water, 0.2 μl of 10 × iPLEX buffer, 0.2 μl of termination mix, 0.041 μl of iPLEX enzyme (Agena), 0.804 μl of 10 μM of extension primer. The single-base extension reaction was carried out at 94°C for 30 s and then 94°C for 5 s, followed by 5 cycles of 52°C for 5 s and 80°C for 5 s, a total of 40 cycles, and then 72°C for 3 min. The reaction mix was desalted by adding 6 mg of cation exchange resin (Agena) and mixed and resuspended in 25 μl of water. The completed genotyping reactions were spotted onto a 384-well spectroCHIP (Agena) using MassARRAY Nanodispenser (Agena) and determined by the MALDI-TOF mass spectrometer. Genotype calling was analyzed using the MassARRAY Typer software version 4.0 (Agena). The primer sequence for COMT Val158Met polymorphism was as follows: forward ACGTTGGATGTAGGTGTCAATGGCCTCCAG and reverse ACGTTGGATGTCATGGGTGACACCAAGGAG. The genotype distributions of the COMT Val158Met polymorphisms in the current sample were Val/Val (*n* = 48; 68.57%), Val/Met (*n* = 19; 27.14%), and Met/Met (*n* = 3; 4.29%), which were in Hardy–Weinberg equilibrium (*x*^2 =^0.39, *P* = 0.53). Given the limited number of Met/Met genotypes, similar to previous studies (Zhang et al., 2016), Met/Met and Val/Met were pooled in the subsequent analyses.

### Statistical Analysis

Statistical analysis was performed using SPSS 18.0 software. A series of preliminary analyses were conducted: 1) G^∗^Power (Version 3.1.9.2) (Faul et al., 2009) was used to determine the appropriate sample size. 2) Outlier detection (Cook’s distance) and normality test (Kolmogorov–Smirnov tests) were performed to ensure the robustness of the results. 3) Pearson correlation analyses were conducted to ensure that participants’ allocation of hot sauce was not associated with their preference. 4) A series of independent *t-*tests were used to detect possible differences in baseline levels of emotional state and of aggression between excluded and included groups, and between different genotype carriers. 5) In order to confirm that the experimental manipulation was successful, independent *t-*tests and Levene’s test of homogeneity of variance were performed on participants’ emotional state after Cyberball game. Finally, two-way ANOVAs with genotype as between-group variable and social condition (exclusion vs. inclusion) as within-group variable were adopted to assess their main and interactive effects on aggression. Comprehensive Meta-Analysis 3.0 (Borenstein et al., 2005) was conducted to compute the overall effect sizes of the interaction between *COMT* Val158Met polymorphism and social condition.

## Results

### Preliminary Analysis

The Cook distance was calculated to assess whether there was any outlier that may distort the outcome, and results indicated that the largest distance obtained in the present study was 0.26, which means that no influential data points should be deleted. The Kolmogorov–Smirnov tests showed that all of the measures of aggression, including hostility, aggressive motivation, and aggressive behavior, differ statistically from a normal distribution. Therefore, data were normalized by using natural logarithm transformation to ensure the accuracy of the following ANOVAs. The results of the Pearson correlation analyses indicated that the amount of hot sauce participants chose to allocate was not significantly associated with their hot sauce preference in any session (*r* = −0.05∼0.09, *P*s > 0.05). The independent *t-*tests also revealed that there were no differences in any initial statements (emotional state, level of aggression), ameliorating concerns over confounding results obtained during exclusion and inclusion [*t*(68) = −0.83∼0.65, *P*s > 0.10] or between different genotype carriers (*t*(68) = −1.75∼1.05, *P*s > 0.05). To confirm whether the experimental manipulation was successful, we examined the difference in the emotional state during social exclusion and inclusion after Cyberball game: Independent *t-*tests showed that the participants in the exclusion group felt more angry and ignored after the experimental manipulation [the first measurement: *Mexclusion* = 2.97, *SD* = 0.86, *Minclusion* = 3.80, *SD* = 0.96, *t*(68) = −3.80, *P* < 0.001; the second measurement: *Mexclusion* = 3.03, *SD* = 0.95, *Minclusion* = 4.00, *SD* = 0.73, *t*(63.54) = −4.79, *P* < 0.001] than those in inclusion group, which meant that the experimental manipulation was effective. Levene’s test of homogeneity of variance indicated that almost all the above-mentioned *t-*tests were in accord with the assumption of equal variances (*F* = 0.01∼2.52, *P* = 0.12∼0.97) with the exception of the last one about the second measurement of emotion state after manipulation (*F* = 4.58, *P* < 0.05).

### Effects of *COMT* Gene Val158Met Polymorphism and Social Exclusion and Inclusion on Aggression

**FIGURE 1:**
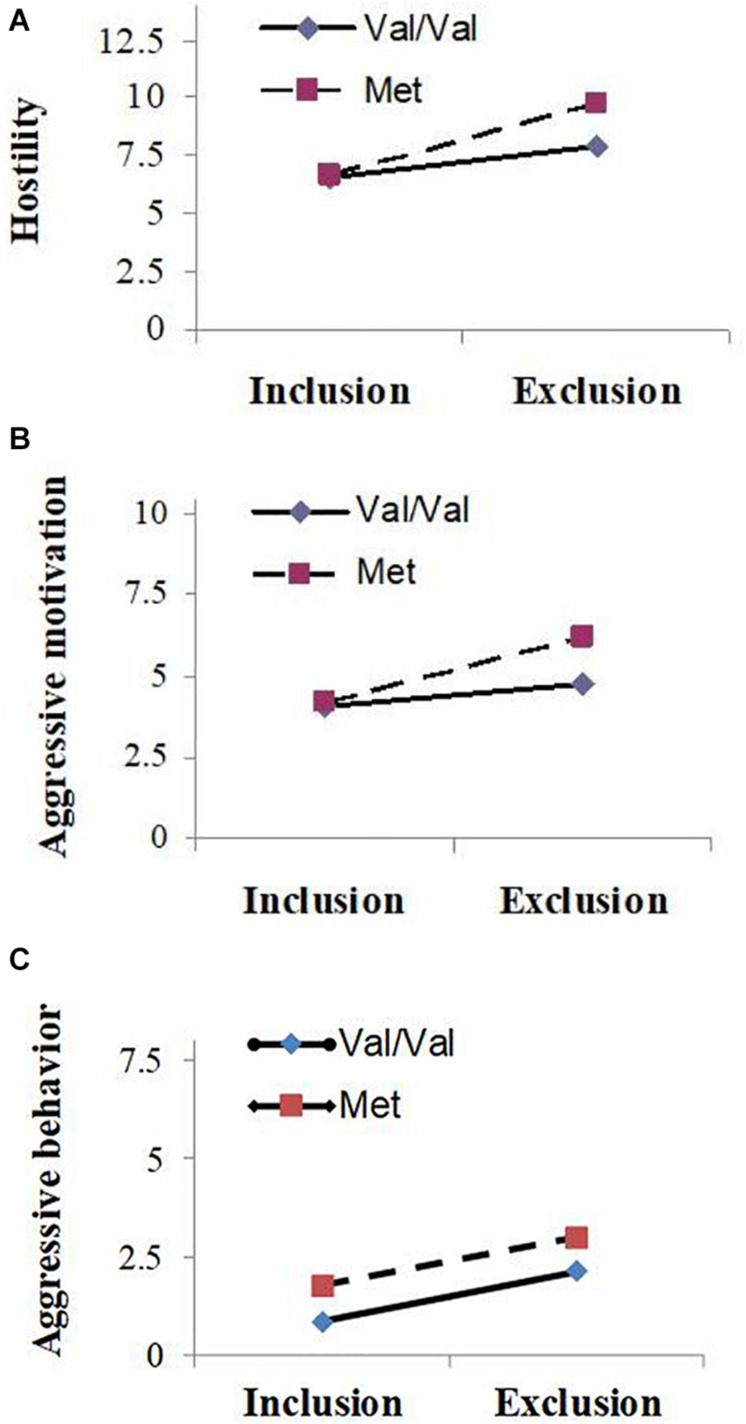
Mean value of hostility **(A)**, aggressive motivation **(B)** and aggressive behavior **(C)** as a function of experimental condition (social exclusion vs. social inclusion) and Catechol-*O*-Methyltransferase (*COMT*) gene Val158Met polymorphism (Val/Val carriers vs. Met carriers).

Means and standard deviations of hostility, aggressive motivation, and aggressive behavior scores are presented in Table 1. ANOVA revealed that the main effects of genotype on hostility (*F*_1,68_ = 4.36, *P* = 0.04, partial $\eta_{\text{p}}^{2}$ = 0.06) and aggressive motivation were significant (*F*_1,68_ = 5.53, *P* = 0.02, partial $\eta_{\text{p}}^{2}$ = 0.08): carriers of the Met allele (Val/Met and Met/Met) showed significantly more aggression than those of the high-activity Val/Val allele; however, a main effect of *COMT* Val158Met on aggressive behavior was not observed (*F*_1,68_ = 2.00, *P* = 0.16, partial $\eta_{\text{p}}^{2}$ = 0.03). As predicted, the main effects of experimental manipulation were also significant (*F*_1, 68_ = 18.06 ∼ 48.36, *P* < 0.001, partial $\eta_{\text{p}}^{2}$ = 0.21 ∼ 0.42): participants showed higher levels of hostility, aggressive motivation, and aggressive behavior under the excluded condition than under the included condition. And as expected, the *COMT* Val158Met polymorphism and the experimental social condition had a significant interactive effect on hostility (*F*_1, 68_ = 5.21, *P* = 0.03, partial $\eta_{\text{p}}^{2}$ = 0.07) and aggressive motivation (*F*_1, 68_ = 4.20, *P* = 0.04, partial $\eta_{\text{p}}^{2}$ = 0.06), although such interactive effect on aggressive behavior was not found (*F*_1, 68_ = 0.007, *P* = 0.86, partial $\eta_{\text{p}}^{2}$ < 0.001). Simple effect analyses showed that for carriers of Met allele, they behaved with significantly higher hostility (*F*_1, 68_ = 31.10, *P* < 0.001) and aggressive motivation (*F*_1, 68_ = 17.98, *P* < 0.001) in the social exclusion condition than the social inclusion condition, or in other words, Met allele carriers exhibited significantly lower hostility and aggressive motivation in the social inclusion condition (M_hostility_ = 6.68, *SD* = 1.56; M_aggressive motivation_ = 4.18, *SD* = 0.50) than exclusion condition (M_hostility_ = 9.73, *SD* = 3.49; M_aggressive motivation_ = 6.23, *SD* = 3.61). For carriers of Val/Val genotype, they also showed significant differences in hostility (*F*_1, 68_ = 17.36, *P* < 0.001) and aggressive motivation (*F*_1, 68_ = 6.79, *P* = 0.01) under different social conditions (hostility: M_exclusion_ = 7.94, *SD* = 2.51, M_inclusion_ = 6.52, *SD* = 1.24; aggressive motivation: M_exclusion_ = 4.75, *SD* = 1.62, M_inclusion_ = 4.06, *SD* = 0.25); however, the differences were more pronounced for Met allele carriers (see Figures 1A–C).

**TABLE 1:** Descriptive statistics for hostility, aggressive motivation, and aggressive behavior.

Variable			Hostility		Aggressive motivation		Aggressive behavior	
					
		*n*	Mean	SD	Mean	SD	Mean	SD
Exclusion	Val/Val	48	7.94	2.51	4.75	1.62	2.13	2.88
	Met	22	9.73	3.49	6.23	3.61	3.00	3.25
	Total	70	8.50	2.95	5.21	2.50	2.40	3.00
Inclusion	Val/Val	48	6.52	1.24	4.06	0.25	0.86	1.55
	Met	22	6.68	1.56	4.18	0.50	1.77	2.67
	Total	70	6.57	1.34	4.10	0.35	1.15	2.00

### Internal Replication Analysis and Meta-Analysis

In order to test the robustness of the above-mentioned findings, an internal replication analysis was conducted by randomly splitting the whole sample into two subsamples. Similar interactive effects between *COMT* Val158Met polymorphism and the experimental social condition were only found in Subsample 1: (hostility: *F*_1, 37_ = 7.47, *P* = 0.01, partial $\eta_{\text{p}}^{2}$ = 0.17; aggressive motivation: *F*_1, 37_ = 3.97, *P* = 0.05, partial $\eta_{\text{p}}^{2}$ = 0.10; aggressive behavior: *F*_1, 37_ = 0.04, *P* = 0.84, partial $\eta_{\text{p}}^{2}$ = 0.01), but not in Subsample 2 (hostility: *F*_1, 29_ = 0.40, *P* = 0.50, partial $\eta_{\text{p}}^{2}$ = 0.01; aggressive motivation: *F*_1, 29_ = 0.62, *P* = 0.44, partial $\eta_{\text{p}}^{2}$ = 0.02; aggressive behavior: *F*_1, 29_ = 0.28, *P* = 0.60, partial $\eta_{\text{p}}^{2}$ = 0.01). The findings from simple effect analyses in Subsample 1 were also replicated. Specifically, compared with participants with Val/Val genotypes (hostility: *F*_1, 37_ = 8.74, *P* = 0.01; aggressive motivation: *F*_1, 37_ = 2.59, *P* = 0.12), those with Met alleles were more sensitive to the change of experimental social condition (hostility: *F*_1, 37_ = 29.57, *P* < 0.001; aggressive motivation: *F*_1, 37_ = 12.79, *P* < 0.01).

A meta-analysis was performed using Comprehensive Meta-Analysis 3.0 (Borenstein et al., 2005) to compute the overall effect sizes of the interaction between *COMT* Val158Met polymorphism and social condition as prior studies (Bakermans-Kranenburg and Van IJzendoorn, 2011; Cao et al., 2018) did. The results indicated that the differences in the combined effect sizes for the impact of social condition on hostility and aggressive motivation between Met carriers and their counterparts were significant (hostility: *Q*_contrast_ = 4.77, *P* = 0.03; aggressive motivation: *Q*_contrast_ = 4.03, *P* = 0.04), with carriers of the Met allele were more sensitive to social condition than those of Val/Val genotype (hostility: for met carriers, Hedges’s *g* = 1.13, *P* < 0.001, 95% CI = 0.61, 1.64; for Val/Val genotype, Hedges’s *g* = 0.51, *P* = 0.001, 95% CI = 0.22, 0.80; aggressive motivation: for Met carriers, Hedges’s *g* = 0.85, *P* < 0.001, 95% CI = 0.39, 1.31; for Val/Val genotype, Hedges’s *g* = 0.30, *P* = 0.04, 95% CI = 0.02, 0.58). However, the difference in the combined effect sizes for aggressive behavior between two genotype groups was not observed (*Q*_contrast_ = 0.000, *P* = 0.99). The Q-statistic test indicated that the two subsamples used herein were homogeneous, *Q* (*df* = 1) < 0.109, *P* > 0.74, *I*^2^ = 0.0%.

## Discussion

We have reported the first empirical study using a mixed experimental design to scrutinize the interactive effect of *COMT* gene Val158Met polymorphism with social exclusion versus inclusion on aggression. Our study extends current work by indicating that *COMT* gene Val158Met polymorphism indeed plays a moderating role on the effects of social exclusion versus inclusion on hostility and aggressive motivation but does not work in a way of “all or none.” The pattern of these findings remains relatively robust across the internal replication analyses and meta-analysis.

Regarding the issue of who (Val vs. Met carriers) was more sensitive to environmental influences, we were intrigued to find that both Val/Val homozygote and Met alleles carriers showed differences in the feelings of hostility and aggressive motivation under conditions of social exclusion versus social inclusion, but these differences were more pronounced for Met allele carriers. This was partially in line with previous work that has demonstrated that the low-activity Met alleles of *COMT* gene were more susceptible to the adverse or supportive environments (Laucht et al., 2012; Thompson et al., 2012; Zhang et al., 2016). Evidence has been provided that Met carriers showed more engagement bias (Gong et al., 2013) and increased activation in the amygdala as well as medial prefrontal regions to negative emotional stimuli (Drabant et al., 2006; Williams et al., 2010); these predispositions to emotional dysregulation would cause Met carriers more likely to be influenced by social exclusion and then exhibit high levels of aggression. This is consistent with the notion of the “aggression cascade” model, which indicates that the occurrence of aggression is associated with gene, environment, and epigenetic interaction involved with induced neuronal deficit and fluctuant neurotransmission (see Cupaioli et al., 2020 for a review).

In seeming contrast to prior studies, our findings showed that the response of Val/Val carriers also varied with environmental conditions. According to the evolutionary perspective, different *COMT* gene variants should have their own unique role in adapting to the environments. The warriors-versus-worriers hypothesis further posits that high-activity Val alleles, an ancestral form that can break down dopamine more efficiently and have better stress resiliency, might have an advantage of dealing with unfavorable or stressful situations (warrior strategy), while Met alleles, a mutant form with less efficient neurotransmission, appear to have an advantage of coping with complex conditions such as tasks of memory and attention (worrier strategy) (Stein et al., 2006; Tuvblad et al., 2016). These above-mentioned viewpoints and our findings in the present study imply that there might be no real plastic or susceptible genotypes, and *COMT* Val158Met polymorphism does not work in an all-or-none way, which means that only one genotype is sensitive to the environment while the other is not. It might well be the case that distinct genotypes react to the same environment at different degrees or react particularly to different environments. Future studies could design at least two distinct experimental conditions, such as complex cognitive tasks and aversive stimuli to further observe their performance of different genotypes carriers under different situations.

Given that the association between environmental stimulation and aggressive behavior can be moderated by self-control (Crescioni and Baumeister, 2009; Esposito et al., 2017), it is not surprising that interactive effects were only observed in the emotional and motivational domains instead of the behavioral domain. This is especially relevant in the current study in which participants were undergraduate students who very not particularly aggressive and would be expected to possess a greater capacity for self-control. This is not to say that aggressive behavior has no genetic underpinnings. Future work is needed on more diverse samples, including female undergraduate students and pathologically aggressive individuals (with conduct disorder, juvenile delinquency, or convicts) to determine the generalizability of the findings reported here in male undergraduate students. It would also be valuable in the future to explore the moderating effect of self-control on the association between environments and aggression.

It is noteworthy that although aggression is a complex phenotype and has polygenic origins, it is still useful to focus initially on specific key genes. One example is the *COMT* gene, which has been shown to play a particularly crucial role in the dopamine regulation of prefrontal region where is scarce of dopamine transporters (Stein et al., 2006). Additionally, relative to multiple-gene design, such as genome-wide research, single gene–environment design is more superior in elucidating the underlying mechanism of aggression, especially under the background that the combined neurophysiological effects of polygenes remain largely unclear up to now.

### Strengths and Limitations

The current study makes an important contribution to literature about genetic mechanism that moderates the individual’s aggression responses to different social environmental conditions. Most notably, the mixed experiment design including between and within factors used herein allows us to better determine whether carriers with the same genotype behave differently under different environmental conditions and offers a methodological framework for developing deeper knowledge in this field of research. Furthermore, instead of only using a measure of aggressive behavior, we assessed the aggression considering motivation, behavior, and hostility, which allows us to better distinguish the *COMT* gene effect in different environmental context on aggression. Thirdly, given that the genetic studies and G × E studies on aggression are often conducted in a clinical population, this study can improve the knowledge in this field of research and allow to lay the groundwork for further psychobiological studies in nonclinical and clinical research context.

Nonetheless, the above-mentioned strengths could not avert limitations that must be acknowledged. Although the power analysis showed that the sample size of this study is adequate for ANOVA, it is still rather limited, especially considering the number of carriers with Met/Met genotype. As most previous studies did, Met/Met and Val/Met genotypes were pooled in the current study, which hindered to check the influence of particular genotype on aggression. Available evidence has shown that the inverted U model exists in the relationship between *COMT* genotype, activity of the prefrontal cortex, and prefrontal dopamine levels and that optimal prefrontal function is achieved with a balanced, moderate dopaminergic activity (Cools and D’Esposito, 2011). Moreover, the undergraduate students enrolled in this study are relatively young and still in adolescence. It was shown that in adulthood, carriers of the *COMT* Met/Met genotype have near optimal dopamine levels, while the Val/Val homozygotes have suboptimal dopamine levels; however, in adolescence, the optimal dopamine levels are present in *COMT* Val/Met carriers and not in the *COMT* Val/Val or *COMT* Met/Met homozygotes (Wahlstrom et al., 2007). Although these students in the present study might be officially adults, it is known that both physical growth and cognitive development can extend into the early twenties. Another concern is that the work presented herein only investigated male undergraduate students, and therefore, caution is advised against generalizability to other groups. Further replications are called for in a larger and more diverse sample.

## Conclusion

The current study, utilizing a mixed experimental design, provides the first evidence of the interaction between *COMT* gene Val158Met polymorphism with social exclusion versus inclusion on aggression in men. Our findings demonstrated that both Val/Val homozygote and Met alleles carriers exhibited differences in the feelings of hostility and aggressive motivation when exposed to conditions of social exclusion versus social inclusion, but these differences were more evident for Met allele carriers. Our new evidence reported here sheds light on the importance of using a mixed experimental design to conduct the research on gene × environment interaction in a deep-going way. One important agenda for future research is to examine the interactive effect of *COMT* Val158Met polymorphism with other environmental conditions on aggression or other psychosocial outcomes using this type of design. Nevertheless, more experimental investigations of G × E interactions are warranted in the future to replicate these findings as well as to clarify the underlying biological mechanism.

## Data Availability Statement

The datasets for this manuscript are not publicly available due to data confidentially and participant privacy. Requests to access the datasets should be directed to MW, wangmeiping@sdnu.edu.cn.

## Ethics Statement

The studies involving human participants were reviewed and approved by the Ethics Committee of Shandong Normal University. Written informed consent to participate in this study was provided by the participants’ legal guardian/next of kin.

## Author Contributions

MW and HL conceived and designed the experiments. MW, PC, and WZ primarily analyzed the data, interpreted results, and drafted the manuscript. AK and WZ provided critical revisions. All authors approved the final version of the manuscript for submission.

## Conflict of Interest

The authors declare that the research was conducted in the absence of any commercial or financial relationships that could be construed as a potential conflict of interest.

## Footnotes

1 https://www.snpedia.com/index.php/Rs4680
